# Vitamin D Regulates the Expression of Glucocorticoid Receptors in Blood of Severe Asthmatic Patients

**DOI:** 10.1155/2021/9947370

**Published:** 2021-08-05

**Authors:** Bassam Mahboub, Saba Al Heialy, Mahmood Yaseen Hachim, Rakhee K. Ramakrishnan, Ashraf Alzaabi, Rania Medhat Seliem, Laila Ibraheem Salameh, Sameen Masooma Toor, Fathelrahman Salem Shendi, Ola Mohamed Al Ali, Basil Khalid Safarini, Wafa Taleb Erabia, Rabih Halwani, Qutayba Hamid

**Affiliations:** ^1^Sharjah Institute for Medical Research, College of Medicine, University of Sharjah, Sharjah, UAE; ^2^Rashid Hospital, Dubai Health Authority, Dubai, UAE; ^3^Department of Basic Medical Sciences, College of Medicine, Mohammed Bin Rashid University of Medicine and Health Sciences, Dubai, UAE; ^4^Meakins-Christie Laboratories, The Research Institute of McGill University Health Centre and the Department of Medicine, McGill University, Montreal, QC, Canada; ^5^Zayed Military Hospital, Abu Dhabi, UAE; ^6^Prince Abdullah Ben Khaled Celiac Disease Chair, Department of Pediatrics, Faculty of Medicine, King Saud University, Saudi Arabia

## Abstract

**Purpose:**

Vitamin D (VitD) deficiency is a significant public health concern in many areas around the globe and has been associated with many immune-mediated diseases, including asthma. Severe asthma has been linked to a decreased glucocorticoid receptor (GR) ratio (GR-*α*/GR-*β* ratio), indicating steroid hyporesponsiveness. Using a combination of *in silico* and *in vivo* approaches, we aimed to explore the immunomodulatory effect of VitD on asthmatic patients diagnosed with hypovitaminosis D.

**Methods:**

*In silico* tools were used to identify the regulatory effect of VitD supplementation on GR genes. We measured the expression levels of GR-*α* and the inactive isoform, GR-*β*, in the blood of adult asthmatics diagnosed with hypovitaminosis D before and after VitD supplementation. Moreover, the blood levels of inflammatory cytokines associated with asthma severity were determined.

**Results:**

Using an *in silico* approach, we identified specific genes commonly targeted by VitD as well as corticosteroids, the mainstay of asthma therapy. NR3C1 gene encoding GR was found to be significantly upregulated on Th2 CD4 cells and NK cells. Interestingly, blood expression level of NR3C1 was lower in severe asthmatics compared to nonsevere asthmatics and healthy controls, while the blood level of VitD receptor (VDR) was higher. Upon VitD supplementation of severe asthmatic patients, there was a significant increase in the blood levels of GR-*α* with no change in GR-*β* mRNA expression. VitD supplementation also suppressed the blood levels of IL-17F and IL-4.

**Conclusion:**

VitD may enhance steroid responsiveness by upregulating the expression of steroid receptor GR-*α*.

## 1. Introduction

Vitamin D (VitD) deficiency is a public health concern affecting all age groups and up to 80% of the population in certain countries [1]. Hypovitaminosis D is prevalent in the Middle East and North Africa (MENA) [2], and VitD level is suboptimal in the residents of sunny United Arab Emirates (UAE) [3]. In addition to its well-established role in calcium and bone metabolism, the potential roles of VitD in cancer development, cardiovascular, and chronic lung diseases are being increasingly appreciated [4]. The increased awareness of VitD deficiency and related health problems have made vitamin D supplementation a routine action plan, albeit without laboratory confirmation in some cases leading to a higher risk of exogenous hypervitaminosis D [5]. Black and Scragg were among the first to show a positive correlation between VitD status and pulmonary function [6]. VitD deficiency is associated with features of asthma, such as decreased lung function, increased airway remodeling, and airway hyperresponsiveness [7]. Several reports have suggested the association of VitD levels with the severity of asthma [8]. Different cross-sectional studies further indicated that low serum VitD in patients with mild-to-moderate asthma is associated with poor asthma control, more exacerbations, reduced lung function, and increased medication use [9–11]. Inverse relationships have also been reported between serum VitD levels and airway remodeling, IgE, and eosinophil numbers, as well as airway hyperresponsiveness. Interestingly, Gupta et al. demonstrated a role for active VitD in correcting IL-10 levels in a pediatric population of moderate and severe therapy-resistant asthma [12]. On the other hand, experimental studies demonstrated that vitamin D receptor- (VDR-) deficient mice fail to develop airway inflammation (decreased infiltration of lymphocytes and eosinophils) and experimental allergic asthma [13].

Steroid resistance, as documented in cases of severe asthma, is associated with increased expression of the glucocorticoid receptor- (GR-) *β*, which is an alternative splicing isoform and dominant-negative regulator of GR-*α* [14]. The abundance of GR-*α* mRNA is higher than GR-*β* mRNA in all normal tissues and cells. Normally, the default splicing pathway leads to GR-*α*, while the alternative splicing pathway that leads to GR-*β* is minimally activated [15]. The latter pathway, however, seems to be upregulated in severe asthmatic patients [16]. In chronic inflammatory diseases like asthma, upregulated blood levels of inflammatory cytokines can participate in glucocorticoid resistance and impair GR function by modulating its translocation, DNA binding, and GR phosphorylation status.

As data from observational studies suggested a better control of asthma following restoration of VitD levels, we hypothesized that correcting VitD levels in adult asthmatic patients could enhance steroid responsiveness by regulating the blood GR-*α*/GR-*β* ratio.

## 2. Materials and Methods

### 2.1. *In Silico* Identification of Top Genes Regulated by Vitamin D Supplementation

We used Comparative Toxicogenomics Database to identify the top genes influenced by vitamin D supplementation in experiments. One hundred and five genes with at least five documented interactions with VitD were identified. Moreover, to explore if the identified genes shared common pathways, Enriched Ontology Clustering for the identified genes was performed using Metascape (a web-based tool used for comprehensive gene list annotation and analysis resource) [17].

### 2.2. *In Silico* Determination of NR3C1, NR3C2, and VDR mRNA Expression Levels in Immune Cells

Using the publicly available “Database of Immune Cell Expression, Expression quantitative trait loci (eQTLs) and Epigenomics,” the NR3C1, NR3C2, and VDR mRNA expression levels in different immune cells were explored.

### 2.3. *In Silico* Determination of the mRNA Expression Levels and Correlations of NR3C1, NR3C2, and VDR in the Blood of Asthmatic Patients Compared to Healthy Controls

We extracted the expression profile of blood from the publicly available U-BIOPRED dataset (GSE69683), which included a sizable number of participants and their detailed clinical information. From a total of 498 participants, data from nonsmokers, which included 87 healthy controls, 77 patients with moderate asthma, and 246 severe asthmatics, were collected while those from 88 smoking severe asthmatics were excluded. Gene expression profiling of blood from these groups was analyzed, and the gene expression of GRs (NR3C1 and NR3C2) and VDR was determined.

### 2.4. Patients

A double-blinded, randomized, placebo-controlled study of VitD supplementation on 54 asthmatics with VitD deficiency was performed. Moderate-to-severe asthmatics between 18 and 65 years of age with clinician-diagnosed asthma and 25-hydroxyvitamin D3 (25D3) levels less than 20 ng/mL at the screening visit were recruited at the Rashid Hospital, Dubai, UAE, and the Zayed Military Hospital, Abu Dhabi, UAE. The patients' hospital electronic records were retrieved, and subjects were excluded if they had any previous VitD supplementation, had any other respiratory disease or comorbid conditions, or were smokers. According to the American Academy of Family Physicians guidelines, participants received 50,000 IU of VitD orally or placebo weekly over 8 weeks. Subjects were blinded to treatment and allocated to receive a VitD dose or placebo by the randomization schedule.

### 2.5. Ethics Approval and Consent to Participate

The study protocol was approved by the Institutional Ethics Committee at both Rashid Hospital and Zayed Military Hospital (MRC-04/2012-06) in accordance with international standards (Declaration of Helsinki), and written informed consent was obtained from all participants. Participants had consented to the use of their collected data and samples.

### 2.6. mRNA Expression and Cytokine Expression in Blood

In line with the U-BIOPRED study, whole blood was collected in PAXgene tubes (Qiagen, Germany) for the isolation of cellular RNA. Blood specimens were assessed for mRNA expression of glucocorticoid receptors (GR-*α* and GR-*β*) using qRT-PCR. Bioplex multiplex immunoassay (Bio-Rad, CA, USA) was used to assess the protein expression of IL-17A, IL-17-F, IFN-*γ*, IL-4, IL-5, and TNF-*α* in serum obtained from patients before and after VitD treatment.

### 2.7. Statistical Analysis

Standard student's *t*-tests and one-way ANOVA were performed to test for statistical significance between data groups using GraphPad Prism 8 (GraphPad, San Diego, CA, USA). *p* < 0.05 was considered significant.

## 3. Results

### 3.1. The Top Vitamin D Regulated Pathways Are Involved in Steroid Response

To identify VitD targeted enriched pathways, we analyzed Comparative Toxicogenomics Database. Interestingly, the top enriched pathways regulated by VitD were involved in several metabolic and inflammatory processes, including response to steroids and lipopolysaccharide as well as interleukin-4, interleukin-13, and AGE-RAGE signaling (Figure S1). Many of these VitD targeted pathways were also targets of dexamethasone. These included pathways involved in inflammation, proliferation, and proapoptosis, besides others (Figure S1).

### 3.2. VDR, NR3C1, and NR3C2 Expression Levels in Different Types of Immune Cells

**Figure 1:**
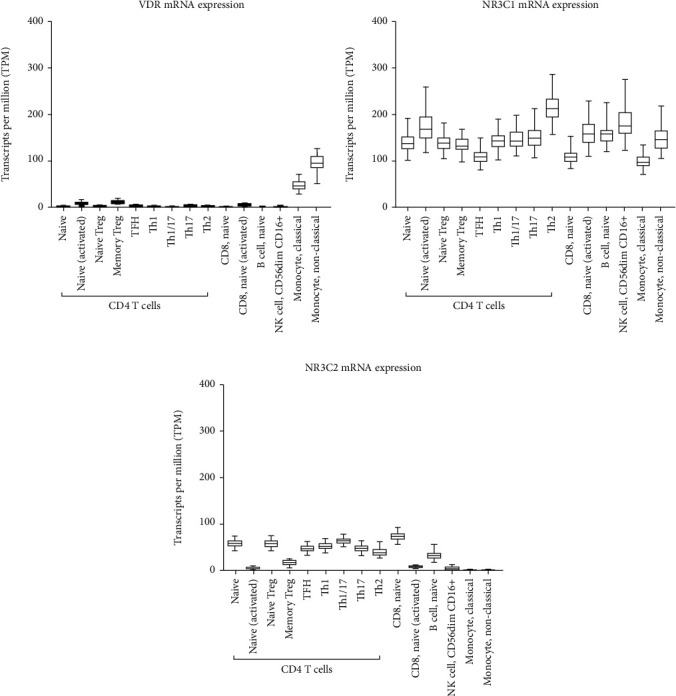
VDR, NR3C1, and NR3C2 mRNA expression levels in immune cells. Data was extracted using the publicly available “Database of Immune Cell Expression, Expression quantitative trait loci (eQTLs) and Epigenomics.”

The level of expression of GR encoding gene NR3C1 (nuclear receptor subfamily 3 group C member 1), mineralosteroid receptor encoding gene (NR3C2) [18], and VDR was then determined in different immune cells. NR3C1 had a significantly higher level of expression compared to VDR and NR3C2 in different types of immune cells (Figure 1). Among the various immune cells, its expression was the highest on Th2 CD4 cells (215.9 TPM). Classical (94.5 TPM) and nonclassical monocytes (46.7 TPM) had the highest VDR expression among all inflammatory cells. Moreover, the expression of all three genes was higher in nonclassical monocytes compared to classical ones (2.0, 1.49, and 1.21 folds, respectively). Naïve CD8 T cells (74.1 TPM) and Th1/17 CD4 cells on the other hand (64.7 TPM) had the highest NR3C2 mRNA expression among all immune cells. Notably, activation of T cells seemed to induce the expression of VDR and NR3C1, but not NR3C2 genes. For example, activated CD4 T cells expressed higher VDR and NR3C1 (3.95 and 1.25, respectively) but lower NR3C2 (0.11-fold), compared to naïve CD4 T cells. Similarly, activated CD8 T cells expressed higher VDR and NR3C1 (10.6 and 1.47, respectively) but lower NR3C2 (0.12-fold), compared to naïve cells (Figure 1).

### 3.3. VDR and Steroid Receptor Genes Are Differentially Regulated in Asthma Relative to Severity

**Figure 2:**
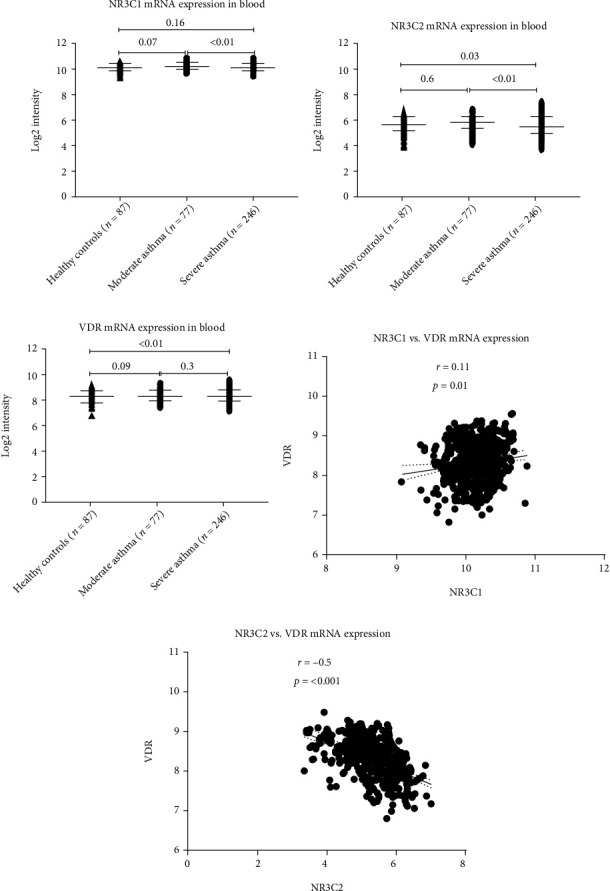
NR3C1, NR3C2, and VDR mRNA expression levels in the blood of subjects with severe asthma, moderate asthma, and nonasthmatics. Data was collected from the U-BIOPRED study (GSE69683).

Our *in silico* analysis demonstrated activated T cells to upregulate VDR, while treatment with VitD suppressed the proliferation of these cells, as well as their ability to produce IFN-*γ* and IL-17 cytokines [19]. The level of expression of VDR in the blood of asthmatics and its association with the expression of steroid receptors relative to disease severity is largely unknown. To investigate that, we analyzed the expression profile of blood from the publicly available dataset (GSE69683) which included a sizable number of participants and their detailed clinical information. A total of 498 participants, of which 87 were healthy controls, 77 with moderate asthma, and 246 nonsmokers with severe asthma, were included from the U-BIOPRED study. The differential expression of NR3C1, NR3C2, and VDR genes in the blood of participants from all groups was then determined. Patients with moderate asthma had higher levels of NR3C1 mRNA expression compared to healthy controls, although not to a significant extent (log2 intensity 10.17 ± 0.29; *p* = 0.07) (Figure 2(a)). When compared to moderate asthmatics, the expression of this gene, however, was significantly downregulated in severe asthmatics (log2 intensity 10.14 ± 0.23 versus 10.26 ± 0.21; *p* < 0.01). NR3C2 mRNA expression was also significantly downregulated in severe asthmatics compared to moderate asthma (log2 intensity 5.397 ± 0.727 versus 5.62 ± 0.5011, *p* < 0.01) and to healthy controls (log2 intensity 5.575 ± 0.5894 versus 5.397 ± 0.727, *p* = 0.03) (Figure 2(b)). Moderate asthmatics showed higher NR3C2 mRNA expression than healthy controls, although the difference was not significant. On the other hand, VDR mRNA expression was significantly upregulated in severe asthmatics compared to healthy controls (log2 intensity 8.386 ± 0.46 versus 8.206 ± 0.44, *p* < 0.01), but not to moderate asthmatics (Figure 2(c)). Interestingly, NR3C1 mRNA expression correlated positively (Spearman *r* = 0.11, 95%confidence interval = 0.02007 to 0.1988, and *p* = 0.01) (Figure 2(d)), while NR3C2 expression correlated negatively (Spearman *r* = −0.55, 95%confidence interval = −0.5724 to -0.438, and *p* < 0.0001) with the levels of VDR expression in blood (Figure 2(e)).

### 3.4. Supplementation of Vitamin D Selectively Favours the Upregulation of GR-*α* Receptor

**Figure 3:**
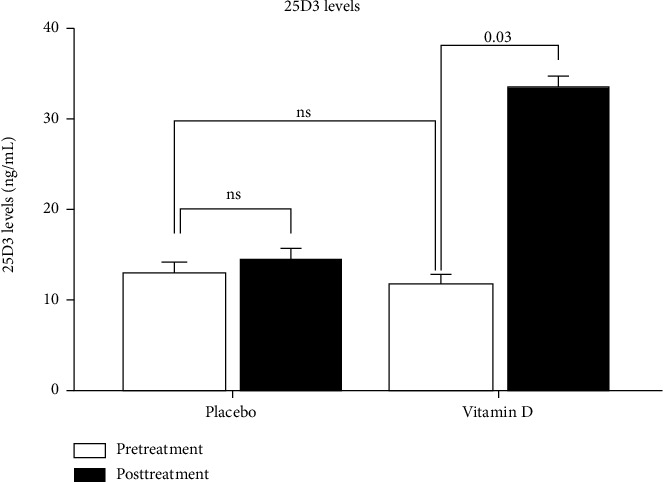
Pretreatment and posttreatment levels of serum vitamin D (25D3) in patients who received vitamin D or placebo for eight weeks. Asthmatic patients with vitamin D deficiency were divided into 2 groups. 23 patients received 50,000 IU of vitamin D orally over 8 weeks, while 22 patients received placebo. Vitamin D levels were measured in the blood of these patients before and after the treatment.

To validate the *in silico* findings, a double-blinded, randomized, placebo-controlled study was conducted to test the effect of VitD supplementation on 54 vitamin D-deficient asthmatics. Moderate-to-severe asthmatics between 18 and 65 years of age who were diagnosed with asthma and with VitD (25D3) levels less than 20 ng/mL at the screening visit were recruited at two hospitals in UAE (Rashid Hospital, Dubai, UAE, and Zayed Military Hospital, Abu Dhabi, UAE). The patients were divided into two groups, an experimental group and a placebo group. The baseline serum levels of VitD in the experimental and placebo groups were comparable (11.74 ± 0.77 ng/mL and 12.96 ± 1.29 ng/mL, respectively). The experimental group received 50,000 IU of VitD via oral supplementation weekly for eight weeks [20]. Following treatment, a 2.84 ± 0.01-fold increase in blood VitD levels (33.34 ± 2.04 ng/mL) was observed (Figure 3). The level of VitD in the placebo group did not change (14.45 ± 1.61 ng/mL) (Table 1 and Figure 3). This result indicated that the regimen used was effective in correcting VitD deficiency.

**Figure 4:**
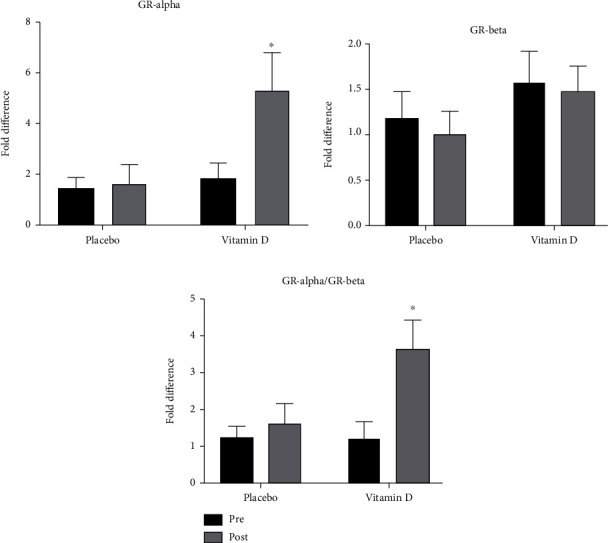
Vitamin D treatment increases the expression of GR-*α* with no effect on GR-*β*. Asthmatic subjects were treated with 50,000 IU of vitamin D orally or placebo for eight weeks. Blood specimens obtained prior to and following 8 weeks of treatment were analyzed for mRNA expression of (a) GR-*α* and (b) GR-*β* using RT-qPCR, and the ratio of (c) GR-*α*/GR-*β* was calculated. ^∗^*p* < 0.05.

We next determined whether VitD supplementation affected the gene expression of steroid receptors, GR-*α* or GR-*β*. Correction of VitD levels led to a significant increase in the expression of GR-*α* when compared to the baseline levels. A significant increase was observed following treatment with VitD compared to pretreatment levels (1.89 ± 0.56 and 5.34 ± 1.40, respectively; *p* < 0.05) (Figure 4(a)). Moreover, there was a significant increase in GR-*α* expression in patients who received VitD compared to those who received the placebo control (5.34 ± 1.40 versus 1.64 ± 0.76-fold; *p* < 0.05). Interestingly, no change in GR-*β* expression was observed following treatment with VitD (1.49 ± 0.27 versus 1.30 ± 0.25) (Figure 4(b)). Relatively, the GR-*α*/GR-*β* ratio increased significantly following VitD supplementation compared to pretreatment levels (3.58 ± 0.83-fold; *p* < 0.05) or patients who received a placebo (Figure 4(c)).

### 3.5. Vitamin D Supplementation Reduced the Blood Levels of IL-17 and IL-4 Cytokines in Asthmatic Patients

**Figure 5:**
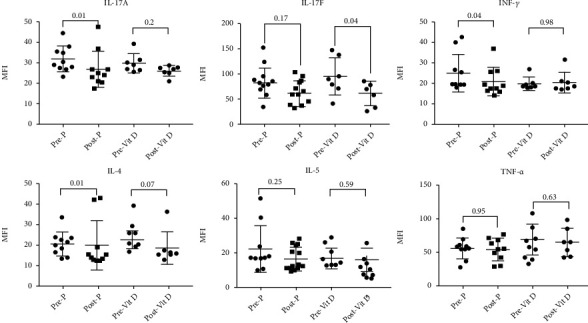
Cytokine profiles of pre- and postvitamin D treatment in the blood of severe asthmatics with vitamin D deficiency. Cytokine levels were measured in the blood of asthmatic patients before and after treatment with 50,000 IU of vitamin D or placebo using Bioplex assay, *n* = 23 for the vitamin D-treated group and *n* = 22 for the placebo group.

The effect of VitD supplementation on the blood levels of asthma-related proinflammatory cytokines was then determined (Figure 5) [21]. Blood levels of IL-17A, IL-17F, IFN-*γ*, IL-4, IL-5, and TNF-*α* were determined before and after VitD supplementation using ELISA assay (Figure 5). VitD preferentially suppressed the blood levels of IL-17F cytokine (*p* = 0.04) without affecting IL-17A, while there was no significant difference in the placebo group (*p* = 0.17). A decrease in blood IL-4 cytokine levels was also observed following treatment, however not to a significant level (*p* = 0.07). No significant effect of VitD supplementation was observed on the rest of the cytokines tested.

## 4. Discussion

This study highlights the role of VitD in glucocorticoid resistance, a significant feature of severe asthma. To our knowledge, this is the first study to determine such an effect of VitD supplementation on glucocorticoid receptor expression in adult asthmatics.

Epidemiological studies have provided early evidence of potential immunomodulatory effects of VitD [22, 23]. VitD binds to the VDR, a member of the superfamily of nuclear receptors expressed by all immune cells. This leads to alterations in the binding of VDR to vitamin D response elements (VDREs) and consequent changes in the transcription of VDRE-regulated genes [24]. By controlling gene expression through the VDR, 25D3, the active metabolite of vitamin D, could limit tissue inflammation [25]. Of interest, multiple lines of evidence have suggested a link between VitD and asthma, including genetic studies showing associations between VDR polymorphisms and asthma [26]. Several studies have reported polymorphisms of the VDR, which subsequently influenced asthma and allergy susceptibility [27, 28]. Animal studies in various disease models have also demonstrated the potent inhibition of proinflammatory chemokine synthesis upon vitamin D treatment [29–31]. Moreover, vitamin D may exert a protective modulatory effect on inflammation by reducing Th17 cytokines in peripheral blood mononuclear cells (PBMCs) and increasing the number of regulatory T cells [32]. The anti-inflammatory effects of vitamin D also include the reduction of TNF-*α*-induced CCL5 and CXCL10 production by airway smooth muscle cells [33].

Using an *in silico* approach and by assessing the transcriptomic signatures of large asthmatic cohorts, we observed a significantly high level of expression of the gene-regulating GR-*α* and GR-*β* receptors, NR3C1, in most of the inflammatory cells tested. Th2 cells had the highest level of expression of NR3C1 compared to other inflammatory cells, which may explain the effective control by steroids of Th2 immune responses, a dominant feature in mild-to-moderate asthma. Interestingly, the expression of this steroid receptor coding gene in the blood of severe asthmatic patients was significantly lower than those of moderate asthmatic patients, which may explain the steroid hyporesponsiveness observed in severe conditions. VDR blood levels were higher in severe conditions, and a positive correlation was observed between VDR and NR3C1, but not NR3C2, which may suggest a possible regulatory association between them. Moreover, the enhanced expression of VDR in the blood of severe asthmatics may explain the observed *in vivo* response to VitD treatment.

To explore how vitamin D may regulate the response to steroids during asthma, we assessed the expression levels of glucocorticoid receptors, GR-*α* and GR-*β*, in VitD-deficient asthmatic patients before and after vitamin D supplementation. Interestingly, correcting VitD levels enhanced the GR-*α*/GR-*β* ratio by upregulating the levels of GR-*α* without affecting GR-*β* levels. VitD supplementation may selectively favour the upregulation of the default splicing pathway leading to an overall increase in GR-*α* expression, with no effect on the alternative splicing pathway, responsible for GR-*β* expression. This is in line with a previous study that reported a significant decrease in the expression of GR-*β* upon vitamin D treatment in subjects with low vitamin D level [34]. By increasing the expression of GR-*α*, VitD may hence rescue the steroid insensitivity encountered in severe asthmatics. Therefore, this study highlights the role of VitD in improving the response to steroids in severe asthmatics suffering from steroid resistance.

We also investigated the effect of VitD supplementation on the expression of proinflammatory cytokines involved in asthma pathogenesis, including Th2 cytokines (IL-4 and IL-5) and Th17 cytokines (IL-17A and IL-17F). Eight weeks of VitD treatment significantly suppressed the expression of IL-17F compared to pretreatment (Figure 5). Although the role of IL-17F in asthma pathogenesis is well documented [35], the mechanism regulating its expression is not very well understood. Recently, the suppression of IL-17F, but not of IL-17A, was found to protect against colitis by inducing Treg cells [36]. A similar mechanism could be involved in the observed anti-inflammatory effect of VitD. We have also observed a decrease in IL-4 blood levels following VitD supplementation, however not to a significant level. Being a key cytokine in asthma pathogenesis, the observed VitD-induced suppression of IL-4 levels may significantly contribute to the efficiency of VitD in controlling asthma pathogenesis. A previous report showed that VitD improved steroid-induced IL-10 production. VitD, however, did not affect IL-10, IL-13, or IL-17A production by PBMCs of moderate and steroid-resistant asthmatic patients [37], similar to what we observed in our cohort.

Some of the limitations of our study include the relatively small sample size. We were unable to follow up with a substantial proportion of our recruited patients after 8 weeks of VitD or placebo treatments as the patients failed to respond to our follow-up requests. This significantly impacted our sample size resulting in a pilot proof-of-concept study.

In conclusion, restoring VitD levels may improve the response of severe asthmatic patients to steroid treatment by enhancing the expression of GR-*α*. Our results highlight the importance of maintaining normal levels of vitamin D in controlling chronic airway inflammation.

## Figures and Tables

**Table 1:** Clinical characteristics of patients who received vitamin D or placebo for eight weeks.

Variable	Total	Placebo	Vitamin D
Total	54	20	34
Male	19 (35%)	4 (20%)	15 (44%)
Female	35 (64.8%)	16 (80%)	19 (55.8%)
Age (years)	38.64 ± 1.90	40.60 ± 2.97	37.45 ± 2.48
Body mass index	32.27 ± 0.92	32.32 ± 5.52	32.32 ± 1.28
Pretreatment 25D3 levels (ng/mL)		12.96 ± 1.29	11.74 ± 0.77
Posttreatment 25D3 levels (ng/mL)		14.45 ± 1.61	33.34 ± 2.04

## Data Availability

All data generated or analyzed during this study are included in this manuscript.

## Supplementary Materials

Supplementary Materials A supplementary figure file is included with this manuscript. Figure S1: functional enrichment, interactome analysis, gene annotation, drug signature, and membership search for the top 105 vitamin D target genes.
