# Supplementary material for: Vitamin D Regulates the Expression of Glucocorticoid Receptors in Blood of Severe Asthmatic Patients
Source: J Immunol Res. 2021 Aug 5;2021:9947370. doi: 10.1155/2021/9947370 (PMC8363450; doi:10.1155/2021/9947370)
Supplement: Supplementary Materials — A supplementary figure file is included with this manuscript. Figure S1: functional enrichment, interactome analysis, gene annotation, drug signature, and membership search for the top 105 vitamin D target genes. [file 9947370.f1.docx]

**Vitamin D Regulates the Expression of Glucocorticoid Receptors in Blood of Severe Asthmatic Patients**

Bassam Mahboub^1,2^, Saba Al Heialy ^3,4^, Mahmood Yaseen Hachim^1^, Rakhee K. Ramakhrishnan^1^, Ashraf Alzaabi^5^, Rania Medhat Seliem^2^, Laila Ibraheem Salameh^1,2^, Sameen Masooma Toor^5^, Fathelrahman Salem Shendi^2^, Ola Mohamed Al ali^2^, Basil Khalid Safarini^2^, Wafa Taleb Erabia^2^, Rabih Halwani^1^, Qutayba Hamid^1,4^


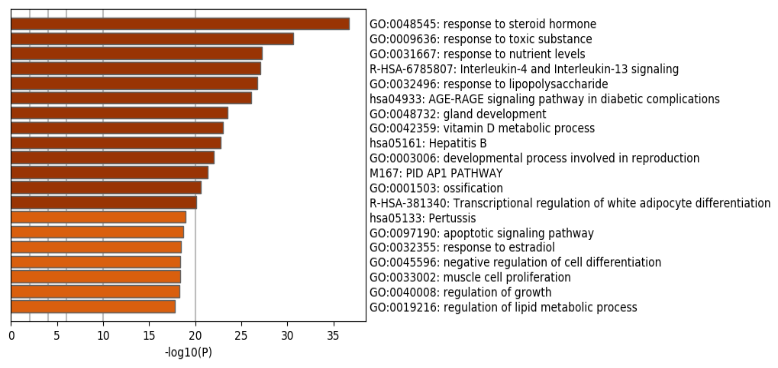


**Figure S1: Functional enrichment, interactome analysis, gene annotation, drug signature, and membership search for the top 105 vitamin D target genes.**
